# Trusting Others During a Pandemic: Investigating Potential Changes in Generalized Trust and Its Relationship With Pandemic-Related Experiences and Worry

**DOI:** 10.3389/fpsyg.2021.698519

**Published:** 2021-08-26

**Authors:** Siri Thoresen, Ines Blix, Tore Wentzel-Larsen, Marianne Skogbrott Birkeland

**Affiliations:** ^1^Norwegian Centre for Violence and Traumatic Stress Studies, Oslo, Norway; ^2^Department of Psychology, University of Oslo, Oslo, Norway; ^3^Department of Psychology, Bjørknes University College, Oslo, Norway; ^4^Centre for Child and Adolescent Mental Health, Eastern and Southern Norway, Oslo, Norway

**Keywords:** generalized trust, pandemic (COVID-19), worry, strain, European Social Survey, perceived health risk

## Abstract

Generalized trust, the belief that most other people can be trusted, has positive consequences for health and wellbeing. An increased sense of community is often seen in times of crisis or disaster, but it is unclear whether this is the case in the COVID-19 pandemic. The objectives of the current study were to assess whether generalized trust increased in an early pandemic phase compared to pre-pandemic levels, and whether trust was lower in individuals who felt particularly threatened or burdened in the pandemic. We compared levels of generalized trust in a population-representative Norwegian sample (*n* = 1,041) with pre-pandemic levels from the European Social Survey (ESS). Age- and gender-adjusted expected scores were compared to observed scores, using weighted data. Secondly, we tested whether indicators of pandemic-related strain, perceived health risks, or pandemic-related worry were associated with a lower level of generalized trust. This cross-sectional study was conducted in an early opening-up phase (May, 2020). The observed levels of generalized trust in an early pandemic phase did not differ significantly from expected levels based on pre-pandemic measures. Higher trust was found among individuals who reported personal experience with the COVID-19 disease (tested positive, admitted to hospital, or lost someone to the disease). Pandemic-related worry and a high perceived health threat were both associated with a lower level of generalized trust. These results indicate that personal experiences with the COVID-19 pandemic could influence trust in others, although this link may be context-dependent. Generalized trust is considered to be an important asset in society, and promote health and well-being. As the pandemic evolves, there is a risk that we may lose, or a chance that we could gain, trust, with potential consequences for our health.

## Introduction

Generalized trust, the belief that most other people can be trusted, is thought to positively influence individuals and society in a multitude of ways (Dinesen, 2012). High trust is associated with better physical and mental health, increased cooperation, well-being, and satisfaction with life (Kawachi et al., 2008; Miething et al., 2020; Zhang, 2020). Although generalized trust is conceptualized as a rather stable factor, major events may have the power to initiate changes in our general evaluation of other people. In this paper, we propose that the current COVID-19 pandemic has impacted trust in the general population and investigate whether trust is particularly affected by pandemic-related strain, worries, and risk perception.

Generalized trust is a phenomenon that has been investigated within multiple scientific disciplines, and there is no uniform agreement about the conceptualization and operationalization of the construct. In survey studies, trust is often defined as the expectation that others will behave with goodwill, keep promises, and avoid doing harm, in other words, a default expectation of people’s trustworthiness (Glanville et al., 2013). Generalized trust refers to expectancies toward other people (strangers or people in general), and is differentiated from institutional, or political, trust (e.g., trust in the police, the justice system, politicians), and from trust in particular individuals (such as family, friends, and acquaintances) (Bjørnskov, 2007; Glanville and Paxton, 2007). Generalized trust is believed to influence behavioral tendencies on an individual and a community level and to promote prosocial behavior and cooperation (Van Lange, 2015). Thus, a high level of trust is desirable both for the individual and for society. Nevertheless, optimal trust may not be the same as maximal trust. A very high level of trust can render an individual or a community vulnerable to deception or exploitation, unless combined with realism or skepticism. This has been called the dark side of trust (Neal et al., 2016).

Whether generalized trust is malleable or resistant to change is a matter of controversy (Dinesen, 2012). Basic trust is first acquired in interactions with reliable caregivers (Rotter, 1980). Thus, early life experiences lay the foundation for a belief that other people can be trusted. This developmental perspective aligns with personality theories, in that both conceptualize generalized trust as a trait that will predict behavior across contexts and experiences, and will determine how new experiences are evaluated. In contrast to institutional trust and trust in specific people, generalized trust is thought to be less fragile as it does not depend as much upon reciprocity (Uslaner, 2015). Generalized trust is therefore considered to be a fairly stable feature of society (Nannestad, 2008). On the other hand, social learning theory postulates that evaluations of other people’s trustworthiness derive from personal and local experiences throughout life (Hardin, 2002). Thus, new experiences, particularly extraordinary or highly emotional events, will shape future expectations about others (Glanville and Paxton, 2007).

The stability of generalized trust has been supported by several empirical investigations (Bjørnskov, 2007; Uslaner, 2008; Bauer, 2015), and challenged by others. A study that tested the two theoretical predictions found support for the social learning theory model, demonstrating that adult experiences did influence trust (Glanville and Paxton, 2007). Additionally, research on immigrants from a low-trust to a high-trust society shows that experiences in a new context affect trust (Dinesen, 2012). Thus, the empirical evidence seems to support both stability and change in generalized trust, indicating that trust may be shaped both by early socialization and later experiences. Generalized trust has been found to be low in individuals with low education and a poor financial situation (Alesina and La Ferrara, 2002).

The COVID-19 pandemic is a slow-motion disaster with worldwide long-term consequences for health, well-being, and economic conditions. When confronted with threatening situations, humans primarily protect themselves and their next of kin, but research has pointed out that acts of mutual aid frequently occur (Mawson, 2005). Humans are vulnerable as individuals, and external threats may stimulate togetherness and connectedness for protection. Experiencing a common faith may strengthen a sense of community (Sibley et al., 2020), and an expectation that one might need help from others in the future also seems to increase trust in others (Cassar et al., 2017). Thus, we can hypothesize that threatening situations, such as the COVID-19 pandemic, may increase generalized trust.

Changes in relationships to other people have been observed in the aftermath of several previous disasters. Following the 2011 earthquake and tsunami in Japan, researchers documented an increase in a feeling of solidarity and trust in others (Hommerich, 2012), and earthquakes have been linked to increased social cohesion in Chile (Calo-Blanco et al., 2017). In the wake of the 9/11 attacks, researchers found that Americans drew closer, not only to friends and loved ones, but also to their fellow citizens (Morgan et al., 2011), although not necessarily to all ethnic and religious groups (Disha et al., 2011). Similarly, Cassar et al. (2017) identified increases in prosocial behavior in areas struck by the 2004 tsunami in Southeast Asia. They argue that the positive effect that living in a tsunami village had on trust occurred due to the experience of receiving help from others. However, not all studies have found an increase in trust following disasters (Fleming et al., 2014). In the COVID-19 pandemic, the countermeasures have likely reduced interactions with other people in the community. According to Putnam, such a reduction in everyday activities, in other words less “bowling together,” will lead to a loss of social capital, including trust in others (Putnam, 2002). On the other hand, a sense of a common destiny, the need to help each other, and the actual experience of helping and receiving help from other people, may increase trust during the COVID-19 pandemic.

Disasters may not promote increased trust for every group in a society (Kang and Skidmore, 2018). Dissatisfaction or discontent with other people’s behavior in the pandemic could erode trust (Kye and Hwang, 2020), perhaps especially for people who are at risk of getting seriously ill, or perceive the health threat as particularly relevant to themselves. Exposure to traumatic events has been associated with low trust (Alesina and La Ferrara, 2002). Additionally, pandemic-related unemployment, economic hardships, and potential social polarization could lead to increased inequality in society, which may result in decreased trust (Bjørnskov, 2007; Brück et al., 2020) among individuals who carry the heaviest burden of the outbreak and the countermeasures.

The pandemic has spurred a massive global research initiative to understand the consequences of the disease threat and the countermeasures for individuals and for society. Risk factors for individual distress related to the pandemic include young age, female gender, financial strain, and unemployment, increased risk for getting seriously ill if catching the virus, psychiatric illness, and extensive worry about the pandemic (Achdut and Refaeli, 2020; Xiong et al., 2020; Blix et al., 2021). Scholars have argued that social capital and social bonds in communities may shape the course of the pandemic (Borgonovi and Andrieu, 2020). Research has demonstrated an increase in institutional trust early in the pandemic (Baekgaard et al., 2020), and a relationship between high governmental trust and adherence to social distancing (Gratz et al., 2021). However, the potential impact of the pandemic on trust has not been thoroughly investigated, even though potential changes in trust may have important consequences for cooperation, health, and well-being. To the best of our knowledge, only one study has been conducted on the relationship between the pandemic and generalized trust, and that study identified a modest increase in trust in South Korea (Kye and Hwang, 2020). One other study found that pandemic-related stress and worry impacted trust in institutions, family, and neighbors (Brück et al., 2020).

The current study attends to this knowledge gap by investigating the potential impact of the pandemic on generalized trust. We propose that generalized trust will increase in the general population during the pandemic, but that the opposite dynamic will occur in people who feel particularly threatened, either because they are in a risk group for the disease or unemployment, perceive their risk to be particularly high, or have a high level of health worry.

In the current study, we pursue the following research questions: (1) Did generalized trust increase in the community in the COVID-19 pandemic compared to pre-pandemic levels? (2) Was generalized trust lower in individuals who carry a heavy burden in the pandemic, in the sense of experiencing pandemic-related strain, perceiving their health risk to be high, or having a high level of pandemic-related worry?

We investigated these research questions in a presumed representative sample of the general Norwegian population by comparing levels of generalized trust during the pandemic with pre-pandemic levels among individuals of the same age and gender in a previous survey. Secondly, we examined whether indicators of pandemic-related strains, perceived health risk, or pandemic-related worry were associated with a reduced level of generalized trust.

## Materials and Methods

### Design

This study was designed as a cross-sectional web survey which was fitted to compare levels of trust in the pandemic with previous levels of trust measured in the European Social Survey. The web survey was performed by the data collection agency Kantar/Gallup, Norway in their panel consisting of approximately 46,000 participants. The panel was constructed to represent the Norwegian general population. Recruitment to the panel is done by probability sampling, not self-recruitment. Sampling is performed based on official statistics from Statistics Norway. The panel is considered to be representative of the Norwegian “internet population” (everyone who has access to the internet), which constitutes about 97% of the total Norwegian population. Data collection was performed during week 21, 2020 (May 18–24). At that time, the COVID-19 situation was described as under control in the Norwegian society, and the government had recently begun easing the countermeasures after a period of approximately two months of lockdown.

### Procedure

The data collection agency approached 2,612 individuals within the panel, stratified on gender, age, education, and area of residence. Potential participants were invited to a survey on well-being and health in the pandemic. The Regional Committee for Medical and Health Research Ethics approved the study (Registration number 133226/2020), and participants consented to participation. Panel members received points for their participation according to the number of minutes estimated to complete the questions. In the current study, estimated to 20 min completion time, participants were rewarded 20 points (equals 20 Norwegian Kroner, 1.9 Euro, or 2.2 USD).

### Participants

In total, 39.9% (*N* = 1,041) completed the survey, 55.8% (*N* = 1,457) did not respond, 2.7% (*N* = 71) started the survey but did not complete it, 1.6% (*N* = 41) clicked on the link to participate but did not confirm their agreement to the terms of the study, and 0.1% (*N* = 2) withdrew from the study. Our study participants did not significantly differ from non-responders in gender or education, but the sample was highly skewed toward older age, with a mean age of 54.1 in responders and 43.3 in non-responders (Blix et al., 2021).

### Tools

*Generalized trust* was measured with three items identical to those used in the European Social Survey^1^ (1) Would you say that most people can be trusted, or that you can’t be too careful in dealing with people? (2) Do you think that most people would try to take advantage of you if they got the chance, or would they try to be fair? and (3) Would you say that most of the time people try to be helpful or that they are mostly looking out for themselves? For each item, participants scored their degree of trust on a scale ranging from 0 (low trust) to 10 (high trust). The mean of the three items was used as an expression of “generalized trust.” Each individual received a mean generalized trust score if they had answered at least two of the three items (99.7%, *N* = 1,038). Cronbach’s alpha was 0.79.

Expected levels of generalized trust were calculated based on data from the European Social Survey (ESS), round 9, collected in 2018^2^. This data material comprises 1,406 participants with ages ranging from 15 to 90. For the purpose of calculating expected trust scores, we used data on the 1,315 ESS participants within the age range 18–89 years, corresponding with the ages represented in our study. Within this age range, 44.3% (*n* = 583) were female, and the mean age was 48.2 (*SD* = 17.3). Cronbach’s alpha for the three generalized trust items in the ESS sample was 0.73. The ESS data are presumably representative of the Norwegian general population, nevertheless, the ESS recommend using weighted data. In the current study, we applied design weights and post-stratification weights to correct for sampling design, sampling error, and non-response bias.

*Sociodemographic factors* age, gender, and education were pre-recorded by the data collection agency. Education was dichotomized into “high” (college or university degree) and “low” (not college or university degree). In addition, participants were asked to evaluate their relative financial situation, as “above average,” “average,” or “below average.” We dichotomized this variable into “below average” and “average or above.”

*Pandemic-related strains* included measures of: (1) pandemic-related job or financial problems, (2) at-risk health condition, and (3) personal experience with COVID-19. Participants reported (yes/no) whether or not they had: (1) lost their job, (2) been temporarily laid off, or (3) experienced financial difficulties because of the pandemic or the countermeasures. Any confirmative answer to these three questions was coded as “Pandemic-related work or financial strain,” vs. no such strain. At-risk health condition (yes/no) was measured with a single question: “Do you have a chronic disease or a health problem with an increased risk of serious disease from COVID-19 (e.g., cancer, heart condition, diabetes)?” Personal experience with COVID-19 was measured by three questions (yes/no), whether or not the participant had: (1) Tested positive for COVID-19, (2) been admitted to hospital because of COVID-19, or (3) knew someone who had died from COVID-19. Any confirmative answer to these three questions was coded as “Personal experience with COVID-19,” vs. no such personal experience.

*Perceived health threat* in the pandemic was measured by two questions from the COSMO study (Betsch et al., 2020): (1) “How likely is it that you will catch the coronavirus?” (mean = 3.45, *SD* = 1.19), and (2) “How serious would it be if you were to catch the coronavirus?” (mean = 4.32, SD = 1.65). Participants responded to both questions on a scale from 1 (low) to 7 (high).

*Pandemic-related worries* the participants were asked to indicate their level of worry on a scale from 1 (not worried) to 7 (very worried) for 12 questions about COVID-related worries adapted from the COSMO study (Betsch et al., 2020). In a previous paper (Blix et al., 2021), we conducted a confirmatory factor analysis, resulting in support for six worry items that are believed to represent an underlying worry factor. We asked participants how much they worried about “losing someone I love,” “becoming seriously ill from the virus,” “infecting others,” “the health system being overloaded,” “not being able to visit people who depend on me,” and “a new outbreak of COVID-19.” Responses to the six questions were averaged to create a composite worry score with Cronbach’s alpha = 0.85 (range 1–7, mean = 3.8, *SD* = 1.2).

### Statistical Analyses

For all individuals in the sample, an expected score was computed as the mean score in the ESS survey for the same age and gender for each single trust item and for the mean trust score. Expected scores were computed both with and without weights. When using weights, weighted means used the weight variables within each combination of age and gender. Confidence intervals for mean differences between actual and expected scores were computed by bootstrapping stratified by sample (the actual and the ESS samples) with 10,000 bootstrap replications. Percentile 95% confidence intervals for the mean differences were computed. The bootstrap procedure included both computation of expected scores and subsequent computation of mean differences. Differences were considered significant if the neutral difference 0 was outside the 95% confidence interval.

Linear regressions were performed to test the associations between generalized trust and pandemic-related strains, threats, and worries. All analyses were adjusted for demographics, i.e., gender, age, education, and pre-pandemic financial situation.

There were no missing values on gender, age, and level of education as these variables were pre-recorded by the data collection agency. Missing on the other independent variables ranged between 0.2 and 3.8% (*n* = 2–40). Only three individuals (0.3%) were not ascribed a mean generalized trust score. Single item missing on the three generalized trust questions ranged from 0.6 to 1.1% (*n* = 6–11).

Descriptive analyses and linear regression analyses were performed in IBM SPSS Statistics 26, while analyses involving expected scores were performed in R (The R Foundation for Statistical Computing, Vienna, Austria, version 4.0.3) with the R package boot for bootstrap analyses.

## Results

The sample comprised 1,040 participants, of which 49.0% (*n* = 510) were female and 35.9% (*n* = 374) had completed a college/university education (a minimum of 16 years of education). Ages ranged from 18 to 89 years, with a mean age of 54.1 (*SD* = 15.9). A perceived lower-than-average financial situation was reported by 12.6% (*n* = 129).

A total of 12.8% (*n* = 129) reported that they had either lost their job (1.9%, *n* = 19), been temporarily laid off (9.9%, *n* = 101), or had experienced financial trouble because of the COVID-19 situation (5.3%, *n* = 54). Although this is the same number of individuals who reported having a lower than average financial situation prior to the pandemic, the overlap was modest and non-significant (15.9% in the lower than average financial situation group vs. 12.2% in the average/above average group, chi square *p*-value = 0.246). A substantial minority (24.9%, *n* = 255) reported that they had a chronic disease or a health problem with an increased risk of serious disease from COVID-19. Only 4.9% (*n* = 50) had personal experience with the pandemic in that they had tested positive for COVID-19 (0.8%, *n* = 8), had been admitted to hospital because of COVID-19 (0.8%, *n* = 8), or knew someone who had died from COVID-19 (4.7%, *n* = 48).

### Level of Generalized Trust

The observed levels of generalized trust (range 0–10) in our sample were 6.4 (*SD* = 2.4) for “Most people can be trusted,” 7.1 (*SD* = 2.2) for “People try to be fair,” 6.3 (*SD* = 2.3) for “People try to be helpful,” and 6.6 (*SD* = 1.9) for the generalized trust mean score.

The differences between observed (O) and expected (E) generalized trust are displayed in Table 1. If O was higher than E, this would indicate an increase in trust from before the pandemic, while O lower than E would indicate decreased trust. Overall, we found no convincing support for the hypothesis that generalized trust in the pandemic had increased compared to pre-existing levels. In unweighted analyses, there were some indications that some aspects of generalized trust were marginally lower in the pandemic. However, when weighting was applied, these differences were very small and non-significant.

**TABLE 1:** Differences between observed (O) and expected (E) generalized trust with 95% confidence intervals (CI).

	**Un-weighted**		**Weighted**	
**Generalized trust items and mean score**	**O-E**	**95% CI**	**O-E**	**95% CI**
Trust 1: Most people can be trusted (range 0–10)	−0.29	−0.42, −0.17	−0.13	−0.27, 0.05
Trust 2: Most people try to be fair (range 0–10)	−0.11	−0.22, 0.01	0.03	−0.10, 0.22
Trust 3: People try to be helpful (range 0–10)	−0.12	−0.23, −0.00	−0.04	−0.16, 0.15
Generalized trust (Mean of trust 1–3)	−0.18	−0.27, −0.08	−0.04	−0.16, 0.13

*O (observed) = reported levels of trust in the current data material. E (Expected) = age- and gender-adjusted expected scores based on pre-pandemic trust measures in the European Social Survey (ESS). A positive value of O-E reflects an increase in trust; a negative value of O-E reflects a decrease in trust. Weighted = Design and post-stratification weights of ESS data.*

### Factors Associated With Generalized Trust

As shown in Table 2, only one of the pandemic-related strains was significantly associated with trust in the adjusted model; increased trust was observed for individuals who reported personal experience with COVID-19 (tested positive, admitted to hospital, or knew someone who died). Perceived probability of catching the virus and perceived health threat if catching the virus were both associated with lower levels of generalized trust when adjusted for sociodemographic variables. However, only perceived health threat if catching the virus was significantly associated with trust in the fully adjusted model. Worrying a lot about the pandemic was also uniquely associated with lower levels of generalized trust.

**TABLE 2:** Associations between demographics, pandemic-related strains, perceived threat and worry, and generalized trust.

	**Single regressions adjusted for demographics^a^**			**Fully adjusted**		
**Independent variables**	**Regression coefficient**	**95% CI**	***p*-value**	**Regression coefficient**	**95% CI**	***p*-value**
**Pandemic-related strains**						
Lost job/financial problems due to the pandemic/the countermeasures	−0.02	−0.37, 0.33	0.931	−0.08	−0.43, 0.27	0.648
At-risk health condition	−0.09	−0.37, 0.18	0.504	0.22	−0.09, 0.52	0.164
Personal experience with covid-19	0.60	0.06, 1.13	0.028	0.70	0.17, 1.24	0.010
**Perceived threat and worries**						
Perceived probability of catching the corona virus	−0.12	−0.22, −0.02	0.017	−0.05	−0.16, 0.05	0.338
Perceived health threat if catching the corona virus	−0.19	−0.27, −0.11	<0.001	−0.17	−0.26, −0.08	<0.001
Pandemic-related worry	−0.21	−0.31, −0.12	<0.001	−0.13	−0.24, −0.02	0.024
**Demographics**						
Gender: Female	−	−	−	0.41	0.18, 0.64	0.001
Age	−	−	−	0.03	0.02, 0.04	<0.001
Education: High^b^	−	−	−	0.19	−0.06, 0.44	0.441
Below average financial situation	−	−	−	−0.89	−1.25, −0.54	<0.001

*Linear regressions with unstandardized regression coefficients, 95% Confidence Intervals (CI), and p-value.*

*^*a*^Separate regression analyses for each independent variable (pandemic-related strains, personal experiences with covid-19, and perceived threat and worries) adjusted for demographics (gender, age, education, and financial situation). Coefficients for demographics are not reported in these analyses, since these coefficients are different in each of these six regression analyses. Adjusted R^2^ in the fully adjusted model = 0.12.*

*^*b*^High education = college or university degree.*

Women reported higher trust compared to men, trust increased with age, and was low in individuals with a poor financial situation. Trust was not significantly associated with education level.

### Aspects of Trust

Table 3 displays adjusted associations between the three single trust items and pandemic-related strains, threats, and worries. Personal experience with COVID-19 was significantly associated with more trust in the categories of “people can be trusted” and “people try to be helpful,” but not “fairness.” Perceiving one’s health risk as high (if the virus were to be contracted) was significantly associated with lower levels on all three aspects of generalized trust. Pandemic-related worry, however, was only significantly associated with a lower score on the item “Most people can be trusted.”

**TABLE 3:** Adjusted associations between Demographics, pandemic-related strains, perceived threat and worry, and the three aspects of generalized trust.

**Pandemic-related strains, threat, and worries**	**Trust 1 most people can be trusted**			**Trust 2 most people try to be fair**			**Trust 3 people try to be helpful**		
	**Coef**	**95% CI**	***p*-value**	**Coef**	**95% CI**	***p*-value**	**Coef**	**95% CI**	***p*-value**
**Pandemic-related strains**									
Lost job/financial problems due to the pandemic/the countermeasures	–0.11	−0.55, 0.33	0.629	–0.24	−0.63, 0.16	0.240	0.11	−0.32, 0.53	0.624
At-risk health condition	0.05	−0.34, 0.43	0.814	0.27	−0.07, 0.61	0.121	0.35	−0.02, 0.72	0.064
Personal experience with covid-19	0.78	0.10, 1.46	0.024	0.45	−0.16, 1.05	0.150	0.86	0.21, 1.51	0.010
**Perceived threat and worries**									
Perceived probability of catching the corona virus	–0.02	−0.12, 0.15	0.826	–0.11	−0.23, 0.01	0.075	–0.06	−0.19, 0.07	0.341
Perceived health threat if catching the corona virus	–0.16	−0.27, −0.04	0.008	–0.15	−0.25, −0.05	0.005	–0.22	−0.33, −0.11	<0.001
Pandemic-related worry	–0.21	−0.35, −0.07	0.003	–0.05	−0.17, 0.08	0.473	–0.13	−0.26, 0.01	0.060
**Demographics**									
Gender: Female	0.15	−0.15, 0.44	0.334	0.57	0.30, 0.83	<0.001	0.51	0.23, 0.80	<0.001
Age	0.02	0.01, 0.04	<0.001	0.03	0.02, 0.04	<0.001	0.04	0.03, 0.05	<0.001
Education: High^a^	0.48	0.16, 0.80	0.003	0.14	−0.14, 0.42	0.327	–0.02	−0.32, 0.29	0.914
Below average financial situation	–0.79	−1.24, −0.34	0.001	–1.15	−1.55, −0.75	<0.001	–0.74	−1.16, −0.31	0.001

*Linear regressions with unstandardized regression coefficients (Coef), 95% Confidence Intervals (CI), and p-value.*

*^*a*^High education = college or university degree.*

## Discussion

In this study, our first aim was to investigate whether generalized trust had increased in the pandemic. Our results did not indicate any overall increase in generalized trust in the Norwegian society in May 2020, compared with pre-pandemic levels. Some previous studies have observed an increased sense of community and trust in the early aftermath of disasters (Morgan et al., 2011; Hommerich, 2012). This phenomenon in disaster recovery has been termed “the honeymoon phase” (DeWolfe, 2000), and is presumably a result of a need to stick together in times of threat, increased prosocial behavior, and a sense of a common destiny (Cassar et al., 2017; Sibley et al., 2020).

The only previous study on generalized trust in the pandemic that we have identified, found a modest increase in generalized trust in South Korea (Kye and Hwang, 2020). Their results differ from our findings of stability in generalized trust. However, it should be mentioned that the measurement of generalized trust differed between the two studies. In the current study we used the three items from the European Social Survey (trustworthiness, helpfulness, and fairness), while the South Korean study used a single item (trust in the South Korean people). Thus, it is not completely certain that the two studies tap into exactly the same phenomenon. Nevertheless, these diverging results may indicate that generalized trust might be impacted differentially depending on local conditions, such as the pre-pandemic trust level, the public adherence to countermeasures, and the local threat level. Further research is needed to clarify whether trust in other people may change with local conditions and over time, as the pandemic unfolds.

As mentioned above, we did not find indications of a change in generalized trust in the community from before to during the pandemic. At first glance, this seems to support the hypothesis that trust is highly stable and robust against new experiences (Uslaner, 2015). However, mean scores may mask subgroup differences, and within our sample, our results indicated that pandemic-related experiences were associated with levels of generalized trust, both positively and negatively. Thus, our results are in line with some previous research which shows that it is possible for trust to be both strengthened and weakened in the disaster recovery process (Kang and Skidmore, 2018). While personal experience with the disease was associated with more trust, higher levels of worry and perceived threat were related to less trust. We found no evidence of a relationship between trust and economic or work-related strains.

Originally, we presumed that generalized trust would be lower in individuals who have carried a heavy burden in the pandemic. Our results show, however, that people who had personal experience with the COVID-19 disease (tested positive, admitted to hospital, or lost someone close) reported a higher level of trust. A potential *post hoc* interpretation may be that these individuals have had positive personal experiences with medical personnel or have received kindness and help from others, which may have resulted in increased trust in other people (Andrabi and Das, 2017). A previous study has also revealed that people who are in need of help in a disaster seem to trust others more (Cassar et al., 2017). We would like to remind the reader that in Norway, the hospitals had not been not stretched beyond capacity, and personal experiences with the disease may have other outcomes in areas with a greater pressure on the health system.

In accordance with our hypothesis, we observed less trust in individuals who perceived their health risk as high or who worried a lot about the pandemic. A similar relationship between worry and trust in family/neighbors and trust in institutions has been identified in a previous study (Brück et al., 2020). Those who feel a particular threat to their health, or who spend a lot of time worrying about a negative future development, may perceive others as a threat and have an attentional bias toward people who do not comply with countermeasures. Perceiving others as threatening and unpredictable may result in a downward adjustment of trust in other people. Keeping an emotional and physical distance from others is not necessarily irrational, as detecting and avoiding threats may be beneficial for survival. Whether threat is associated with protective behavior is, however, debated. Some studies indicate that perceived threat and fear may not be the best predictors of compliance with countermeasures. Rather, perceived self-efficacy, i.e., feeling capable of following advice, seems to motivate protective behavior in the pandemic (Jørgensen et al., 2020).

Although worry and perceived health risk were both uniquely associated with lower trust, these two factors differed in their relationship with the three separate trust items. A high perceived health risk was significantly associated with lower perceived trustworthiness, fairness, and helpfulness of other people. Worry about the pandemic, however, was only significantly related to trustworthiness. Our measure of worry included worries about getting infected oneself, but the majority of items tapped into more social aspects, such as losing someone close, not being able to help significant others, and more general aspects, such as worry about a new outbreak and an overloaded health system. Thus our results may indicate that personal threat relates to a general downward adjustment of trust, and that worry perhaps specifically relates to how much people can be trusted to adhere to important countermeasures in the pandemic. Future research is necessary, however, to replicate and expand on these findings, and to assess if a potential loss of trust will be restored when the pandemic has passed.

Finally, loss of employment and/or financial difficulties due to the pandemic did not seem to affect generalized trust. We would like to remind the reader that the data in this study was collected in May, 2020, in an early opening-up phase, during which countermeasures were eased. Thus, employment loss or financial difficulties may have been perceived as temporary at the time. A previous study investigating the link between institutional trust and unemployment demonstrated large differences between countries. In some countries, this link was strong, in others it was weak, suggesting that the context of these experiences are key to their effect (Brück et al., 2020). The potential impact of these factors may rest upon the general welfare practices in each society; the current study was conducted in a high-income society with social welfare practices that may have somewhat compensated for the marginalization effect of (perceived temporary) unemployment. If, over time, the pandemic brings about a financial recession, increased inequality, and polarization, a long-term outcome might be loss of generalized trust in society (Bjørnskov, 2007).

### Limitations

The present study compared levels of generalized trust in the pandemic with pre-pandemic levels, but the regression analyses were conducted in the cross-sectional data. We used a stratified probability sample from a panel representing “Norway in miniature” according to a set of sociodemographic variables. Our response rate of 39.9% is within the expected range for this type of study. Responders did not differ substantially from non-responders in gender or education, although young people were significantly under-represented, reflecting a trend in survey research that is not specific to this study. Nevertheless, non-responders may have differed from responders in undiscovered ways. It is possible, for example, that engagement in social issues or in the pandemic may have led to increased interest in participation. Furthermore, we used only self-report measures. This is probably not a limitation for measuring generalized trust, for which there are no alternative methods of measurement in survey studies, but other items may have been subject to interpretation by the participant (e.g., “financial difficulties”). We measured trust beliefs (expectancies toward other people), and were not able to investigate trust as a behavior. Participants first responded to questions about pandemic-related stress and strains, secondly to a series of questions into the quality of social relationships. The preceding questions may have affected their responses to the trust items. Moreover, we were only able to compare levels of trust at one time point in the pandemic with pre-pandemic levels, and the development over time could not be investigated. Most importantly, our study was conducted in a specific society at a specific time, and it is likely that the relationships between pandemic-related experiences and trust will differ between localities and over time.

## Conclusion and Implications

In conclusion, we did not observe any overall increase in generalized trust in the early phase of the pandemic in the Norwegian society. The level of generalized trust was, however, higher in individuals with direct experience with the disease and lower in individuals who perceived themselves to be at particularly high risk or who worried a lot about the pandemic. These results indicate that personal experiences with the COVID-19 pandemic may have an important influence on trust in others. Thus, this study lends support both to the notion that generalized trust is established early in life and is fairly stable in society (Nannestad, 2008; Uslaner, 2015), and is, additionally, shaped by personal experiences throughout life, particularly new, extraordinary, and highly emotional events (Hardin, 2002; Glanville and Paxton, 2007).

Research so far in the pandemic has focused mainly on the immediate physical and mental health consequences. But the pandemic represents much more than a health crisis, and may prove to have far-reaching implications for societies at large. In light of the increased risk of future pandemics (Madhav et al., 2018), research on the COVID-19 pandemic is of great value to understand how the health risk and the countermeasures can affect trust. Of particular importance is research that can assess under which conditions trust is affected, for whom, and for how long. A previous study has shown that loss of institutional trust in disaster victims may last for decades (Thoresen et al., 2018), but future research is needed to identify factors that help restore trust when countermeasures are eased. Multi-national studies, such as the European Social Survey, are well suited to answer questions regarding the development of trust over time in different countries, but need to be accompanied by data on individual pandemic-related experiences.

Such research can assist governments in disaster planning and communication strategies, to potentially counteract a loss of trust in society. National and regional governments may encourage helpfulness and pro-social behavior, particularly toward at-risk groups, such as individuals who worry a lot or perceive their health risk as high. Similar strategies may also be implemented in hospitals, health and care services, and educational institutions. Theoretically, personal experience with helping others and receiving help will increase social trust, but there is a need to test the effectiveness of such interventions in the context of pandemics. Generalized trust is considered an important asset for health and well-being. As the pandemic evolves, there is a risk of losing, and an opportunity for gaining, trust, with potential consequences for health. The link between pandemic-related experiences and trust probably depends heavily upon the context. Future trust in society may be stimulated by interventions in the health sector, care and kindness toward those who worry and feel threatened, and welfare benefits for those who become unemployed.

## Data Availability Statement

The datasets presented in this article are not readily available because According to the Ethics committee, we may not share the data anonymously until the personal identity in the original data file is deleted. Requests to access the datasets should be directed to corresponding author.

## Ethics Statement

The studies involving human participants were reviewed and approved by the Regional Committee for Medical and Health Research Ethics, Southern and Eastern Norway. The patients/participants provided their written informed consent to participate in this study.

## Author Contributions

ST was the principal investigator in this study. ST, IB, and MB contributed equally to the design of the study, the data collection, in organizing the data file, and in formulating the research questions. ST had the lead in writing up the manuscript, with significant contributions from IB, MB, and TW-L. TW-L and ST carried out the statistical analyses. All authors have read the manuscript in the submitted form.

## Conflict of Interest

 The authors declare that the research was conducted in the absence of any commercial or financial relationships that could be construed as a potential conflict of interest.

## Publisher’s Note

All claims expressed in this article are solely those of the authors and do not necessarily represent those of their affiliated organizations, or those of the publisher, the editors and the reviewers. Any product that may be evaluated in this article, or claim that may be made by its manufacturer, is not guaranteed or endorsed by the publisher.

## Footnotes

1
www.europeansocialsurvey.org/

2
www.europeansocialsurvey.org/data/country.html?c=norway
